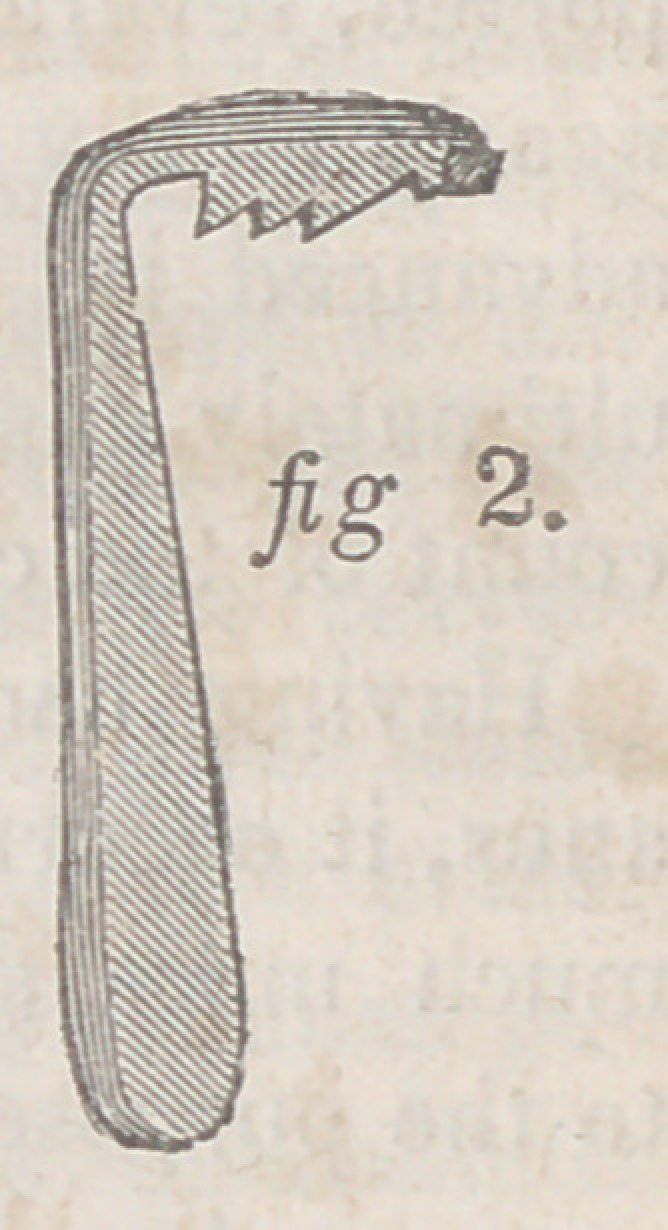# A Monograph on the Nature and Treatment of Artificial Anus

**Published:** 1843-07

**Authors:** Samuel D. Gross

**Affiliations:** Professor of Surgery in the Louisville Medical Institute


### THE WESTERN JOURNAL OF MEDICINE AND SURGERY.

JULY, 1843.

### Art. I.—*A Monograph on the Nature and Treatment of Artificial Anus.* By Samuel D. Gross, M.D., Professor of Surgery in the Louisville Medical Institute.

In the January, February, and March numbers of this Journal, I published an elaborate paper on the Nature and Treatment of Wounds of the Intestines. To that paper the present essay is designed as a supplement. It is believed that this is the first attempt in this country, if not in the English language, to exhibit the subject of Artificial Anus in a connected and methodical point of view, and if it shall have the effect of attracting the attention of the profession I shall consider myself amply compensated for the time and labor it has cost me.

It must be obvious that the term "artificial," applied to this affection, and in vogue among American and British authors, is rather ill chosen. In its etymological sense it merely implies some production of art, as an artificial leg, or an artificial eye; while in surgical language it denotes the effect of some operative or mechanical procedure, as the formation of an artificial pupil. By most of the French writers it has been superseded, in reference to the present topic, by the word "preternatural", and this is unquestionably preferable, in all respects, as it is much more expressive of the true nature of the malady which it is intended to designate. Equally appropriate is the term "accidental," used by some of the continental surgeons. The word artificial, in fact, should be restricted to that form of the affection, in which the abnormal outlet is established mechanically, for the purpose of affording relief when there is some insurmountable obstacle in the rectum, or lower bowel. In employing the term "artificial," therefore, in connexion with the present subject, I am governed rather by the established usage of the profession than by the rules of sound criticism.

Artificial, accidental, or preternatural anus may occur in any part of the abdomen; but, as it is generally produced by gangrene of the bowel, from the pressure which is exerted upon it by hernial stricture, it is by far most frequently met with in the inguinal, scrotal, femoral, and umbilical regions, particularly the first two. For the same reason we find that the small intestine is much oftener involved than the large, which is fixed or attached, while the former is loose, floating, and consequently more liable to protrusion. Occasionally, though rarely, the abnormal anus has its seat in the lumbar region, high up in the iliac, the hypochondriac, or even the epigastric.

Three causes mainly give rise to this affection, namely, strangulated hernia, accompanied with mortification of the bowel, penetrating wounds, and stercoraceous abscess; the frequency of their occurrence being in the order in which they are here enumerated. A blow or kick on the abdomen may so contuse, bruise, or injure the bowel as to lead to the establishment of an artificial anus. Jobert saw a case in which an opening was formed in this way between the ileum and the vagina,* and examples of a similar kind have been witnessed by others.

When the bowel is extensively divided by a sharp instrument, and the wound is managed improperly, or left to itself, the patient either perishes from peritoneal inflammation caused by fæcal effusion; or adhesions take place between the gut and the adjacent parts, and the contents of the tube issue at the external orifice. The latter always happens when this accident is treated in conformity with the method of Palfin, Bell, and Scarpa, who advise the inner wound to be kept in apposition with the outer, by a ligature passed through the mesentery.

Stercoraceous abscesses are induced by various causes; sometimes by ulcerative action, often by external violence, and occasionally by the irritation created by the presence of a foreign body, as a needle or pin, a fish or chicken bone, or a piece of coin. In either case, as soon as the matter is discharged, whether spontaneously or by the efforts of the surgeon, the fæces escape at the abnormal aperture, either wholly or in part, and the patient is affected, not merely as some have pretended, with an intestinal fistula, but with a genuine preternatural anus. Large fæcal accumulations have sometimes been mistaken for this kind of abscess; the knife or lancet has been plunged into them, and the disease in question has been the consequence, or the individual has died from peritoneal inflammation. Artificial anus is occasionally congenital, in which case it is usually seated at the umbilicus.

Mortification, like penetrating wounds, may affect the entire circumference of the bowel, or only a part of it. The extent of the lesion will exert a material influence upon the restorative process, and in this respect the disease might not inappropriately be divided into partial and complete.

The external orifice of an artificial anus exhibits no uniformity in respect to its size and configuration. In many

---

* Maladies du Canal Intestinal, T. ii, p. 95.

cases it is rounded, in some ovoidal, and in most irregular. In its diameter it varies from a few lines to an inch and a half or even two inches; being usually smaller in traumatic cases, or in such as result from penetrating wounds, than in those which are produced by ulceration, abscess, and especially by gangrene. The margins of the opening are thick, bevelled, depressed, or inclined towards the centre, where they are in close contact with the mucous membrane of the two ends of the bowel, the junction between them being indicated by a reddish line; they have a raw, flesh-colored appearance, and are covered with numerous granulations, which are often very painful, and so irritable as to bleed upon the slightest touch. The matter which they secrete, and which is seldom very abundant, does not differ from that of other sores under similar circumstances. In cases of long standing, or where the fæcal discharges are unusually acrid, the edges are very much indurated, inflamed, highly sensitive, and studded with fungous vegetations, some of them the size of a split-pea, or even half a dime. In a third series of cases, perhaps, they are elevated, hard, and almost insensible. The skin in the immediate vicinity of the opening, as well as for some distance beyond it, is red, inflamed, chapped, fissured, excoriated, or ulcerated, and so tender frequently that the patient cannot bear to have it touched, wiped, or washed, however gently this may be done.

The depth of the outer orifice, or the distance between the skin and the bottom of the intestinal aperture, varies from three to twelve lines. It is always less when the disease is produced by a wound than when it is caused by gangrene; much will also depend upon the natural thickness of the wall of the abdomen, and the degree of plumpness or emaciation of the individual. Lallemand met with an instance where the distance between the two points was nearly two inches, and in another, which fell under the observation of Delpech, it was upwards of three inches.*

---

* Dict. de Medicine, T. iii, p. 347.

The external orifice is occasionally multiple, that is, instead of a single opening there are several. In this case there are usually fistulous tracks, which communicate with the main outlet, and sometimes even with each other. Velpeau mentions an instance in which there were not less than five or six distinct apertures, and another, not less remarkable, is related by Dupuytren.* This perforated and cribriform state of the parts is generally produced by some of the stercoraceous matter insinuating itself among the muscular fibres and cellular substance of the abdomen before the margins of the external orifice are sufficiently protected by the new adhesions. An abscess soon forms, preceded by an erysipelatous blush of the skin, and followed by a discharge of purulent matter, almost insupportably fœtid in its character.

The union between the two ends of the bowel and the circumference of the outer orifice is effected through the medium of plastic matter, and constitutes an indispensable element of the disease. The inflammation, preceding and accompanying the effusion, always begins in the serous surfaces of the parts, from which it gradually extends to the other structures, as the mucous membrane, the muscles, cellular substance, and the skin. The plastic matter, soft and glutinous at first, is soon organized, and thus opposes an effectual barrier to the effusion of fæcal matter into the abdominal cavity. Subsequently it undergoes all the changes that lymph experiences, under favorable circumstances, in other situations. The extent of this adhesion varies, in different cases, from half a line to a line; it rarely amounts to half an inch, or, indeed, even the fourth of an inch, and in proportion as it is firm or otherwise will it be able effectually to resist the influence of such causes as have a tendency to separate the gut from the wall of the abdomen. Dupuytren met with two cases in which the union was so feeble that the intestine lost its hold, and the patients died from fæcal effusion.

When the artificial anus supervenes upon strangulated her-

---

* Dict. de Medicine, T. iii, p. 346.

nia, the formation of these adhesions usually precedes the death of the bowel; in the traumatic variety of the affection, on the contrary, they are established after the reception of the injury, and hence the greater frequency of fatal effusion in the latter than in the former. As the adhesions extend only a small distance along the gut, a cul-de-sac is formed, the opening of which looks towards the belly, and into which the abdominal viscera may protrude, so as to complicate the disease.

Immediately around the inner margins of the outer orifice are, as was previously stated, the two ends of the bowel; lying generally side by side, like the tubes of a double-barrelled gun. Each opens by a distinct orifice, of which the upper, in time, becomes much the larger; they are bounded by a sort of villous rim, are irregularly rounded in their form, and are separated from each other by a septum or partition. The upper orifice gives passage to the fæces, and, as it is unprovided with a sphincter muscle, the patient has no control whatever over their escape. Even mechanical means will not always obviate this inconvenience, and the utmost attention to cleanliness does not defend the surrounding parts from the effects of the acrid discharges. The lower orifice, of the same size at first as the upper, is generally very narrow, puckered up, and sometimes even difficult to be found, especially when it has ceased for a long while to receive fæcal matter. The upper opening is temporarily closed when the corresponding extremity of the bowel is touched with a probe or finger, or exposed to a few drops of cold water. When thus irritated it presents very much the appearance of the anus of the horse, the mucous lining being everted and corrugated by the peristaltic action of the muscular fibres.

The two ends of the bowel, at first similar in size, by degrees undergo important changes. The upper continually giving vent to fæculent matter, bile, mucus, and even ingesta, receives a preternatural quantity of blood, and hence generally acquires a considerable increase of volume and strength; its coats are thicker than in the normal state, the muciparous follicles are larger, the lining membrane is of a deeper red, and the peristaltic action is inordinately energetic. The lower extremity, on the contrary, having no longer any active function to perform, falls into a state of atrophy. Its tunics are pale, flaccid, and attenuated, its caliber is considerably diminished, though not obliterated, and its mucous glands are wasted and almost imperceptible. The canal contains a soft, whitish, gelatinous looking substance, which is evidently the product of an imperfect secretion, and which is voided by stool at intervals of two, three, or four months. The intestine, notwithstanding these alterations, still preserves its tubular form, however long the fæces may have been discharged through the abnormal aperture. That this is the fact has been proved by repeated dissections. Thus, Lecat examined the body of a female who had labored under this malady for twelve years, and in whom the inferior portion of the gut, or the part comprised between the natural and artificial outlet, was still pervious, though much contracted. Similar observations have been made by Desault and Dupuytren. The latter opened a patient, two years after the establishment of an artificial anus, and found that the tube not only remained open, but that it had experienced comparatively little diminution. The following case, however, observed by Mons. Begin, of Paris,* shows that the obliteration of the intestine, although extremely rare, is not impossible. The patient was eighty years of age when he died, and for more than half this period he had labored under an artificial anus, seated in the left groin, and communicating with the arch of the colon. The superior extremity of the bowel only opened at the external orifice, and gave passage to the fæcal matter. No aperture, corresponding with the other end, could be discovered either in the cicatrice or in the surrounding parts. The gut itself was converted into a hard, solid, whitish cord, not thicker than a common quill, which

---

* Dict. de Med. Chir. Pratiques, T iii, p. 133.

passed to the left kidney, from which it descended, after several turns, to the anus, increasing somewhat in size as it approached its termination. The inferior part was still pervious, and contained a little whitish mucus; the upper for the length of six or eight inches, next to the abnormal aperture, was completely obliterated; and the intermediate portion was so contracted as scarcely to admit a small probe.

This atrophy or wasting is not confined to the lower portion of the bowel, but often affects the corresponding part of the mesentery and even the lymphatic ganglions. As might be supposed, it is always more marked in old than in recent cases.

In artificial anus, caused by a gangrened rupture, the two ends of the bowel are surrounded and closely embraced by a sort of membranous pouch, to which Scarpa, who first described it, has applied the name of *infundibulum* or *funnel.** It is formed by the prolongation of the peritoneum which constituted the neck of the hernial sac, and varies very much in its shape, dimensions, and direction, its base being at the bowel, and the apex at the skin. It is generally very firm and dense in its structure, and from one to two lines in thickness, according to the extent of the previous inflammation; externally it is intimately united to the margins of the abnormal opening, and internally it presents a smooth villous surface, not unlike that of an old fistulous track. The fæculent matter from the upper orifice is poured into this cavity, and thence, when the artificial anus is closed, it is carried, after describing a half-circle, into the lower end of the canal. This membranous pouch is always wanting when the disease is the effect of a penetrating wound, and occasionally even when it is the consequence of a gangrened hernia: in both cases the gut adheres immediately to the edges of the opening in the muscles and integuments. The most interesting circumstance connected with this funnel-shaped cavity is the influence which it exerts upon the reparative process, or spon-

---

* Treatise on Hernia, Memoir Fourth, p. 288.

taneous cure, which is always so much the more prompt and perfect in proportion as it is larger and longer.

Interposed between the two extremities of the intestine, and formed by the juxta-position of their sides, is the *ridge, septum,* or *partition,* which Scarpa has described under the

*

name of the promontory, and Dupuytren under that of the epéron, spur, or buttress. It consists of two angular or crescentic folds composed each of four lamellæ, of which the inner two are of a serous nature, and firmly united together by plastic matter, for an extent varying from one to six, eight, or even twelve lines. The outer layers are of a mucous character, and are continuous with the lining membrane of the

* *a a,* Opening of the artificial anus, and point of union between the skin and the mucous membrane; *b,* upper end of the intestine; *c,* lower end of the intestine; *d,* the septum, epéron, or ridge, formed by the walls of the two contiguous cylinders; *e e,* parietes of the bowel; *f,* the ligament or cord formed by the mesentery; *g,* the cul-de-sac between the peritoneum of the intestines and of the abdominal walls, into which herniæ occasionally protrude.

tube, of which they form a part. Dividing the bottom of the funnel, where it is situated, into two unequal parts, this septum juts out nearer to the surface of the abnormal opening in proportion as the loss of intestinal substance has been more considerable, and the change in the direction of the tube more marked. It is small, and scarcely perceptible, when the gut has been merely pierced by a wound, or slightly affected by an eschar, but large and prominent, when the lesion, whatever it may be, involves the whole circumference of the canal.* In the former case, the two orifices of the bowel are separated by a kind of gutter or groove, which directs the transit of the fæcal matter from the one to the other, and greatly facilitates the attempts at cure; in the latter, the septum forms a projecting angle or buttress, which conducts the contents of the upper orifice towards the abnormal outlet, and which nothing but art can break down or surmount.

When the two lamellæ of which this septum is composed are viewed posteriorly, or from within the belly, we find that they gradually recede from each other, leaving thus a triangular interval between them, the apex of which corresponds to the point of separation, and the base with the abdominal cavity. The surfaces of these lateral layers, which are, in fact, nothing but the parietes of the affected cylinders of the bowel, are invested by a reflection of the peritoneum, and afford attachment to a process of the mesentery. From the manner in which this membrane is stretched between the spinal column and the concave side of the intestinal convolutions, it follows that it is always more or less dragged on when the gut is protruded from the belly, forming a sort of cord by which the body is inclined forwards, and the tube drawn inwards. A constant traction is thus kept up, which varies in degree in different cases, and which has occasionally been sufficient to destroy the adhesions between the bowel and the wall of the abdomen, causing fatal effusion into the cavity of the peritoneum.† Dupuytren, who has devoted

* Dupuytren, Dict. de Med. and Chir. Pratiques, T. iii, p. 130.

† Dupuytren, Leçons Orales, T. ii, p. 207.—London Medico-Chir. Review, vol. ix, p. 315.

much attention to this subject, states that this tension of the mesentery is continued long after the malady is removed. Several individuals who had been cured of artificial anus, were subsequently re-admitted into the Hotel-Dieu, where they died of other diseases. On dissection it was ascertained, contrary to what might have been expected, that the bowel, instead of being adherent to the walls of the abdomen, was free and unattached; a solid fibrous cord, however, being still stretched between them. This last was only a few inches in length by several lines in thickness, greatly attenuated at the middle, invested by peritoneum, and formed entirely of condensed cellular substance. Had these individuals lived a little longer this band would, doubtless, have been gradually destroyed, and every vestige of the malady ultimately disappeared.

The matter which issues at the abnormal opening varies in its properties according to the length of time it is retained in the bowel, the nature of the food, and the state of the patient's health. Generally speaking it is soft, semi-fluid, or even quite liquid, of a greenish color, and composed of an admixture of fæces, bile, and intestinal secretion, together with ingesta. Its consistence is always less when the artificial anus involves the jejunum or the superior extremity of the ileum than when it affects the lower portion of the small bowel, the cœcum, or the colon. In the former case, too, it has less stercoraceous odor, and occasionally contains a considerable quantity of pancreatic juice. The frequency with which it is voided is materially influenced by the nature and quality of the food, as well as by the manner in which it is prepared, and by the distance which intervenes between the abnormal aperture and the stomach. When the artificial anus is situated near this organ, it commonly passes off within an hour or two after eating, whereas, if it be lower down it may not be voided for five or six hours, or perhaps not oftener than three or four times a day. The evacuations, as was before intimated, are always involuntary, and are generally effected with considerable rapidity, being accompanied with a peristaltic movement of the upper extremity of the gut, and a sort of rumbling noise, especially when there is an escape of air.

The quantity of fæcal matter flowing along the abnormal opening, like its quality and the frequency of its discharge, must necessarily be influenced by a variety of circumstances. Of these the most important are the amount of food, the extent of the intestinal lesion, and the size of the septum between the two ends of the bowel. Most persons laboring under this disease eat voraciously, often, indeed, three or four times as much as they did before; they are always hungry, have an enormous appetite, and are never satisfied. This is particularly the case when the ingesta are retained only for a short time. Hence there is a proportionably large accumulation of fæculent matter, and as this cannot pass from one intestinal orifice into the other, in consequence of the mechanical obstacle interposed between them, most of it, if not all, escapes at the abdominal opening.

The pernicious influence which the brief sojourn of the alimentary matter exerts upon the system is not always so great as might be supposed. Indeed, not a few instances are related in which the patients not only retained their health and strength, but even grew fat. In the generality of cases, however, the effects are quite the reverse. The food is retained too short a period to be properly acted upon by the digestive organs; the function of chylification is impaired; nutrition is carried on imperfectly; the body is emaciated, and there is a proportionable failure of the physical powers. In extreme cases, that is, where the general health is otherwise affected, or where the passage of the aliment is exceedingly rapid, the patient has sometimes perished from inanition.

Another very serious inconvenience to which persons laboring under artificial anus are subject is the protrusion of the extremities of the gut. This often amounts to a real prolapsus, and is liable to occur, no matter what may have been the cause of the disease. It may affect one or both ends, but the upper is more frequently involved than the lower, though the reverse is said to be the case by Boyer, not, however, with any foundation in truth. The extent of the prolapsus varies, in different cases, from three to eight inches; more rarely it amounts to a foot, or even a foot and a half. In its diameter the tumor seldom exceeds two and a half or three inches. It is more or less conical in its shape, contracted at the base, and perforated at the extremity by an irregularly rounded opening. The everted mucous membrane is at first only preternaturally red and vascular; by degrees, however, it becomes thickened, rugose, indurated, and completely hypertrophied. In this respect it experiences the same changes of structure as the villous coat of the rectum in prolapsus of the anus. The swelling, which is commonly much larger in the erect than in the recumbent posture, frequently possesses so little sensibility that it may be touched or handled without pain. At times, however, it is excessively tender, and may then become a source of real suffering, depending more, perhaps, upon the state of the system than upon that of the part immediately concerned. Strangulation of the prolapsed intestine occasionally occurs, and, although the stricture by which it is produced, may generally be easily relieved by an operation, yet in several instances it has terminated fatally. Sabatier, in his Memoir on Artificial Anus, quotes two examples from Puy, a surgeon of Lyons, where death was caused in this way; an instance of a similar kind fell under the observation of Flajani, and another is mentioned by Le Blanc, in the second volume of his "Operations de Chirurgie." In a case narrated by Mons. Veiel,* the division of the stricture did not prevent death.

From this rapid sketch of the nature, anatomy, symptoms, and complications of artificial anus, it is obvious that, in whatever light it be viewed, it must be regarded as one of the most distressing affections to which we are liable. Independently of its filthy and disgusting character, the patient has

---

* Archives Generales de Medicine, 2d series, T. vii, p. 542.

scarcely a moment of comfort of any kind; the skin of the abdomen is constantly fretted and chafed, in spite of the utmost attention to cleanliness, the general health is often seriously deranged, the digestive organs are apt to become impaired, the bowels are frequently racked with colicky pains, the mind is gloomy and despondent, and life itself is a burden. Death is occasionally produced by inanition, or by the laceration of the new adhesions and the consequent effusion of fæcal matter into the peritoneal cavity; but these occurrences are rare, and more generally the patient lives in the manner mentioned, and is ultimately carried off by some other disease. The probability of spontaneous reparation, or a cure by surgical interference, will depend very much upon the seat of the complaint, the nature of the exciting cause, the freedom from complications, and the extent of the septum or partition between the two ends of the gut. It need hardly be stated that the age of the patient and the state of his health exercise considerable influence on the prognosis.

*Treatment.*

The treatment of artificial anus naturally divides itself into palliative and radical. The first consists in promoting the comfort of the patient, by strict attention to cleanliness, preventing too early an escape of the ingesta, and combating such accidents or complications as may arise during the progress of the malady. The radical treatment has for its object the re-establishment of the natural course of the fæces, and the obliteration of the opening in the wall of the abdomen. These topics involve important principles, and therefore require separate consideration.

It is a question which has not yet been definitively settled how soon, after the occurrence of an artificial anus, we are warranted in attempting a radical cure. Several examples are now on record where, by premature interference of this kind, the patients lost their lives. Death, under these circumstances, may be produced by a variety of causes, but the most common is, perhaps, the want of adhesion in the sides of the opening which is made in the epéron or intervening septum, and the consequent escape of fæcal matter into the abdominal cavity. Another source of mischief is the imperfect union between the ends of the bowel and the margins of the abnormal outlet. Indeed, it appears to me that, until this union is fully established, or is so strong and firm as to render it impossible for it to give way under the traction of the enterotôme or the manipulations which are necessary to introduce the seton, it would be highly improper, with a view to a radical cure, to do any thing calculated to jeopard the result by fæcal effusion into the peritoneal cavity. We have no means, unfortunately, of ascertaining how soon the plastic matter, by which the union in question is effected, becomes organized, and capable of withstanding such forces as have a tendency to break up the new connexions. Nevertheless, there is reason to believe, from what we know in regard to the changes which coagulating lymph experiences in other parts of the body, that several months, probably from three to six, are necessary for the purpose. Prior to this period, therefore, I would deem any surgical interference officious und unadvisable, particularly in relation to the enterotôme of Dupuytren, or the modification of this instrument by other practitioners. The method of Desault, of which we shall presently speak, may be advantageously resorted to much earlier, and consequently before the adhesions between the contiguous parts have acquired all the strength of which they are capable.

There is, therefore, a period, unless a spontaneous cure should in the meantime supervene, of several months during which the patient must bear with his loathsome infirmity, and suffer all the inconveniences arising from the effusion of fæculent and other matters. This, however, is not all. The case may be such, as, by its very nature, to preclude the possibility of effecting a radical cure by any means of which we are in possession; or the patient may, from timidity or other causes, be unwilling to submit to an operation of any kind. In either event, it is the duty of his attendant to make his situation as comfortable as practicable.

The first and most important object to be attended to, in a case such as we have imagined, is to prevent the escape of fæcal matter at the artificial anus; or, if this cannot be done, to apply some apparatus for receiving and retaining it. When the disease has been caused by a wound, or when the bowel has been only partially destroyed by gangrene, the former of these indications is generally easily fulfilled by means of a piece of gum-elastic, several lines thick, shaped like a nipple-shield, and large enough not only to cover the external orifice, but to extend some distance beyond it. This, being soft and flexible, readily accommodates itself to the various movements of the body, and answers the purpose much better than leather, tin, brass, or sheet-lead, recommended by some surgeons. It should be retained by a graduated compress and bandage; or, what would be better, a common truss with a broad pad perforated in the centre for receiving the knob on the gum-elastic plate. To derive full benefit from this apparatus it might be so constructed as to have a projection on its posterior surface, carefully fitted into the abnormal opening, which it would thus more effectually close, at the same time that it would prevent the protrusion of the bowel, so liable to occur when the parts are imperfectly supported, or when the patient is in the erect position.

When the fæces can not to be made to pass along the natural channel, in consequence of the inordinate size of the septum between the two ends of the bowel, the patient may generally be rendered very comfortable by wearing an apparatus for their reception and temporary retention. The older surgeons were in the habit of using, for this purpose, receptacles of leather, or horn, which were fastened round the body by bandages of particular construction. These contrivances, however, rarely fulfilled the intention for which they were constructed, as it was found not only difficult to adapt them accurately to the parts, but from the facility with which they imbibed moisture they soon became offensive, and required to be often renewed. The most perfect apparatus of this kind, perhaps, was constructed many years ago, by Juville, a celebrated Parisian truss-maker. It is delineated in his "Traité des Bandages Herniaires," and has occasionally been worn with great benefit. In its construction it is exceedingly complicated, and it is scarcely possible to convey a correct idea of it without a plate. It is composed of a common inguinal truss, the pad of which is made of ivory, and rests upon the margins of the artificial opening. To a hole in the centre of the pad is fitted a tube of gum-elastic, which is furnished with a valve, and directs the fæcal matter to a silver receiver, fastened to the inner part of the thigh. The silver receptacle is of a flattened conical shape; it is three inches in length by two inches and a half in breadth, and may be unscrewed and emptied without disturbing the rest of the instrument. The valve in the tube opens by its own weight when the patient is in the erect position, but shuts when he lies down, and prevents the accumulated fæcal matter from reflowing into the artificial anus. When the apparatus is properly adjusted it is said to answer so well that the patient is able to pursue his ordinary occupation, and to escape the inconveniences arising from the discharge of fæculent and other matter.

If, notwithstanding the use of an apparatus of this kind, the fæces are diffused over the surrounding surface, the utmost attention must be paid to cleanliness. Without this no comfort can be expected. If allowed to remain in contact with the skin, even for a short time, the acrid discharges not only induce pain and irritation, but they render the patient loathsome to himself, and disgusting to his friends. In fact, there is no situation in which a human being can be placed which is more pitiable and distressing, or better calculated to excite our sympathy.

When inflammation arises, either in the part itself, or in the neighboring integuments, it is to be combated by frequent ablutions, emollient poultices, and anodyne fomentations. If it partakes, as it sometimes does, of an erysipelatous character, it may be necessary, in addition to these means, to use leeches and blisters. The callosities which are so apt to form on the surface of the sore should be removed with the knife or scissors; escharotics are to be avoided, as they always give rise to severe pain, and rarely afford much relief. When the skin is ulcerated, fissured, chapped, excoriated, or studded with pustules, it should be thoroughly cleansed with a soft sponge and tepid water, after which it must be anointed with simple cerate, and covered with a slippery-elm or linseed poultice, These dressings are to be continued as long as may be necessary, and renewed twice or three times in the twenty-four hours.

Fistulous tracks, when they exist, must be incised, and their edges pared, to put them in a condition favorable to cicatrization. The cure is sometimes much retarded, if not entirely prevented, by the perforated state of the parts; the skin, perhaps, is indurated and disorganized, and fæcal matter issues at various points, keeping up constant irritation and distress. In such a case it may become necessary to remove the affected structures, bowel and all, with the knife, to reduce them to the nature of a simple wound.

Inanition is seldom to be apprehended when the artificial anus is seated in the large intestine, or low down in the small. In either event, abundant time is usually afforded to the chyliferous vessels for taking up the nutritious portion of the ingesta, and conveying it to the proper receptacles, before the contents of the tube reach the abnormal outlet. When this, however, is situated higher up, the case may present a very different aspect, as the chymous matter may escape too soon to enable the system to be much benefited by it. This circumstance is always known by the soft and lactescent nature of the discharge, by the voracious appetite, and by the progressive emaciation. The patient eats three or four times the accustomed quantity of food, his hunger is never appeased, he is thin and haggard, and has but little strength. To support life, which is occasionally much endangered by this occurrence, the individual must be kept perfectly quiet, the diet must be light, nutritious, and easy of digestion, the irritability of the bowels must be allayed by anodynes, and the outer opening must be well protected with an obturator. In bad and intractable cases, threatening life, advantage might be derived from the use of nutritious injections.

When the bowel becomes prolapsed, as it is apt to do when the opening is not properly closed, the reduction is usually effected with great facility, by placing the patient on his back, and making gentle and well-directed pressure upon the part with the fingers of one hand, while the bowel is held between the thumb and fingers of the other. In more obstinate cases, the replacement may be attempted by systematic compression, while the patient is lying on his back; this will have the effect of emptying the protruded gut of its blood, and will often succeed after other and more simple means have failed. When the reduction is impracticable, the gut should be supported by a well adjusted apparatus, and the patient should refrain from laborious exertion, from laughing and coughing, irregularities of diet, and from every thing tending to increase the swelling. When symptoms of strangulation arise, the most rigorous antiphlogistic measures are to be adopted; blood is to be abstracted, both generally and locally, the patient is to be placed in the warm bath, and cloths, wrung out of tepid water, are to be constantly applied to the affected part. In a word, the case is to be treated precisely as when the strangulation is produced by ordinary causes. When these remedies fail, relief must be attempted by an operation. The patient lying upon his back, near the edge of the bed, the surgeon takes a bistoury which he passes along the fore-finger of the left hand, and makes a free incision through the integuments around the base of the tumor, which is generally sufficient to remove the stricture. Should this, however, be found not to be the case, it will be necessary to extend the incision into the end of the bowel, at its union with the margin of the abdominal orifice. Soon after the stricture is divided, the tension of the part subsides, the fæces flow out externally, the pain disappears, and the gut gradually becomes reducible.

Artificial anus is susceptible of spontaneous cure. Of this numerous examples are on record, and there is scarcely a surgeon, at all extensively engaged in practice, who has not met with cases of it. The fæces, after having passed for weeks or months through the orifice in the abdomen, gradually resume their natural channel, the artificial anus closes up, and at length all that remains is a small cicatrice, indicating the situation of the former injury.

A singular instance of artificial anus, in which a cure was effected during pregnancy, has been related by Dr. Wedemeyer of Hanover.* The woman was thirty-two years of age, and the disease, caused by a gangrened hernia, had existed for seven months, during which it had resisted various methods of treatment. As pregnancy advanced, and the uterus ascended into the abdomen, the fæcal discharge diminished, and passed proportionably along the natural route. Towards the close of gestation, nothing issued at the abnormal opening, except a little pus and serum, and in two months after her accouchement the parts had completely healed.

Before we attempt the radical cure of an artificial anus by any method of treatment, however simple, it is proper that some attention should be paid to the general health of the patient. If this be much deranged, it is obvious that it should be rectified, otherwise the case may proceed badly, or even terminate fatally. The secretions are to be restored; the diet is to be regulated; the bowels must be moved by mild aperients; and any local irritation that may exist is to be combated by frequent ablutions, anodyne fomentations, and emollient poultices. Leeches will seldom be necessary. This preliminary treatment is particularly called for when we wish to put in

---

* American Medical Recorder, vol. xiii, p. 453.

execution the operation of enterotomy, autoplasty, or even the more simple one of the seton.

When the abdominal orifice is too small to admit of the ready application of the enterotôme, the use of the plug, or the passage of the seton, it must be dilated with linen tents or gum-elastic bougies. The foreign substances should not be introduced too frequently, or too forcibly, or for too long a time; and their use should not be attempted until the parts are divested of their tenderness and irritability. By proceeding cautiously in this manner, taking care gradually to increase the size of the tent, the abnormal passage may generally be dilated to the requisite extent within from two to four weeks. When the edges of the track are very thick and callous, the treatment will be greatly expedited by excising them.

After the operation has been performed, the patient must for sometime lie quietly upon his back, with the legs and knees drawn up, to relax the abdominal muscles and prevent undue compression of the parts. The diet must consist of nourishing broths or light soups, soft boiled rice, tapioca, arrow-root, or boiled milk and grated cracker. For his drink he may use demulcent fluids, as gum water, or flaxseed tea; the bowels should be calmed by anodynes, and the natural stools promoted by stimulating enemata. In short, every thing is to be done to avoid inflammation, both in the parts more immediately concerned, and above all in the peritoneum.

Of the various operations that have been devised for the radical cure of this loathsome and disgusting affection, the first that attracts our attention is the suture. The idea of employing enteroraphy is generally supposed to have originated with Lecat. However this may be, it is certain that a female laboring under this infirmity, was under the care of that celebrated surgeon in 1739, and would have been subjected to this treatment had she not become tired of the numerous attempts that had been made to replace the protruded and adherent gut.

The expedient was subsequently carried into effect by a surgeon of the name of Bruns; but instead of paring the edges of the opening, as had been suggested by Lecat, he contented himself with excoriating them with caustic. The case seemed to be going on favorably, when, on the third day, the ligature lost its hold, and the anus began to gape, followed by a discharge of fæcal matter. The patient, unwilling to submit to further trials, was abandoned to his fate.

The next attempt at this species of enteroraphy was made by Liotard;* but the result was not more fortunate. He pared the whole circumference of the abnormal aperture, and approximated the raw edges by two points of suture, aided by a favorable position and proper dressings. On the second day the apparatus was observed to be soiled by fæcal matter, and on exposing the parts the ligatures were found to have cut themselves out. Professor Blandin, of Paris, in a similar case, was equally unsuccessful. Indeed, Judey seems to be the only surgeon in whose hands the expedient has hitherto had a favorable termination: the artificial anus had existed four months, and the cure was complete.†

Desault, in the latter part of the last century, endeavored to cure this disease by compression.‡ He was aware that the chief obstacle to the reparative process was the septum between the two ends of the tube, by which the fæculent and other matters were diverted from their proper channel, and forced out at the preternatural orifice in the wall of the abdomen. The removal of this projecting piece constitutes, therefore, a most important indication in the treatment. To fulfil this, Desault used long linen tents, which he introduced and fixed in the two ends of the gut, taking care to renew them as often as they became soiled and offensive, which generally happened once or twice in twenty-four hours. In this manner he gradually effaced the abnormal angle, and

---

* Diss. Sur. le Traitement des Anus Contre Nature. Paris, 1819.
† Archives Generales de Medicine, T. i, p. 291.
‡ Surgical Works; Translated by Smith, vol. i, p. 306. Philadelphia, 1814.

brought the two cylinders into a straight line; at the same time he dilated the lower orifice, and thus placed it in a more favorable condition for receiving air and fæcal matter. Along with these means he kept the outer opening closed by a well-adjusted compress, for the two-fold purpose of preventing the escape of the fæces, and of forcing them on towards the rectum. When the dilatation was sufficiently advanced and the intervening septum nearly effaced, he discontinued the long tents, and merely retained the external plug, applying it more superficially lest it should interfere with the transmission of the contents of the tube, and so become an obstacle to the restorative process. If it succeeds, the good effects of this method are announced by slight colicky pains and rumbling noises in the belly, followed at first by a discharge of wind, and soon after by fæculent and mucous matter. In proportion as the natural passages are re-established the outer orifice diminishes in size, and the griping subsides. The cure is expedited by a light but nutritious diet, perfect rest, and an occasional enema.

Although this method of treatment has been repeatedly attended with the happiest results, it is obvious that it cannot be employed with a prospect of success in all cases. Of this Desault himself appears to have been fully aware, and he has enumerated the following circumstances as particularly calculated to oppose the cure, or as so many contra-indications: first, where the gut has suffered great loss of substance; secondly, where the intervening septum is too short, prominent, or difficult to be broken down; and thirdly, where the ends of the bowel, one or both, have contracted such firm adhesions externally as to render it impracticable to effect their reduction. In addition to these considerations it may be remarked that the process is generally very tedious, and well-calculated to exhaust the patience both of the surgeon and the sufferer.

The first instance in which Desault employed this mode of treatment is too interesting to be passed over on the present occasion. The case, in fact, has become memorable in the annals of surgery. The following abstract of it is all that my limits will allow me to give.

Francis Vialter, a large, strong, and well-built man, a sailor and native of Moulins, was injured by the bursting of a bomb in May, 1786. The wound occupied the right side, and extended from about two inches above the inguinal ring to the bottom of the scrotum, where it had exposed the testicle. At the upper angle was a sort of appendix, very red, an inch long, and formed by the divided bowel, which retracted into the abdomen, whenever the parts were washed. In this situation an opening was left in the dressings for the discharge of the fæces. After having wandered about for four years, and visited all the principal hospitals of Europe in vain for relief, he entered the Hotel-Dieu of Paris, on the 29th of September, 1790.

The upper end of the bowel, from long exposure to the air, the friction of the clothes, and the contact of fæcal matter, had acquired considerable thickness, as well as density, and was of a conical shape, nine inches in length. Its base, which was somewhat contracted, seemed to proceed from beneath a fold of the skin, just above the inguinal ring; while the apex, inclined backwards, reached to the middle of the thighs, and ended by a narrow orifice, through which the fæces flowed. Nothing had escaped by the natural passage since the period of the injury, except a small quantity of whitish, ropy mucus, at intervals of three or four months. The whole surface of the tumor was red and wrinkled, like a villous membrane. On the outer side of this mass was another protrusion, much smaller but of the same color and consistence, oval in its form, and puckered like the mouth of a purse at the extremity, where it discharged a little serosity. The patient was extremely emaciated, and compelled, by the violent pains he experienced in the abdomen, to bend himself forward when he attempted to walk. An earthen pot, attached to the waist and suspended between the thighs, received the fæcal matter.

To reduce the swelling of the upper protrusion, without which the bowel could not be restored to its natural situation, Desault used a simple roller, with which he covered the whole tumor from below upwards, by spiral and moderately light turns, leaving merely an orifice at the apex for the passage of the fæces. The effect of the treatment was extraordinary; the tumefaction rapidly subsided, and by the fourth day the intestine was in a condition to be replaced. Having accomplished this, he opposed the issue of the excrements by a thick linen tent, three inches long, introduced into the gut, and supported by an inguinal bandage. His idea was to remove this twice a day for the evacuation of the fæces; but in a short time the patient perceived a rumbling noise in the abdomen, accompanied with an acute sense of heat, and wind was soon expelled by the anus. Colicky pains occurred in the rectum, and half a pint of fluid matter was discharged by the natural route. The night following he had a number of evacuations of the same kind, preceded by similar feelings, and which left him somewhat languid in the morning. The passages became gradually more natural, the pains disappeared, and on the eighth day the tent was discontinued, the external opening being closed by a pledget of lint and several compresses, supported by a truss with a broad flat pad.

From this period Vialter rapidly recovered; he regained his flesh and strength, and voided his fæces without pain or inconvenience. A very trivial serous exhalation moistened, without staining, the lint which covered the *fistulous* orifice in the abdomen. Five months after he left the hospital, in attempting to lift a cask on his shoulders, his bandage broke, and the intestines again protruded, to the length of six inches, through the unhealed opening. The same treatment as on the former occasion was adopted with complete success.

A more effectual and less tardy expedient than that of Desault, was proposed, near the close of the last century, by Schmalkalken, a German surgeon. He has given an account of it in his Inaugural Dissertation, "Nova Methodus Intestina Uniendi," published at Wittemberg in

1798. The operation consists in perforating the septum with a seton introduced by means of a curved needle, and allowed to remain long enough to excite adhesive inflammation between the two contiguous cylinders of the bowel. The period necessary for this varies from a week to a fortnight, at the expiration of which the foreign body is withdrawn, and the portion of the partition lying between the outer orifice and the opening of communication excised with a pair of scissors or other suitable instrument. In executing the operation, it is of no little importance that the track made by the instrument is accurately filled by the thread or tape, otherwise fæcal matter might be effused into the peritoneal cavity. The seton ought to be carried as high up as possible, and care taken that it do not embrace a neighboring fold of the intestine.

Whether Schmalkalken ever performed this operation, of which he must undoubtedly be regarded as the inventor, it is impossible for me to say, as I have not before me a copy of the dissertation in which the account of it originally appeared, nor do I find the subject mentioned in any other work. The probability is that he did not; at all events, very little notice of it was taken even in Germany, while the profession of the remainder of the continent of Europe, Great Britain, and the United States appears to have been profoundly ignorant of it. I make this statement for the purpose of showing that the late Dr. Physick, of Philadelphia, who performed a similar operation in 1809, was not aware that it had been previously proposed, and that he is therefore justly entitled to a share of the credit of the discovery.

The case which fell under the observation of Dr. Physick, and which was probably the first of the kind treated by the seton, was that of John Exilius, a Swedish sailor, nineteen years of age, who was admitted into the Pennsylvania Hospital in October, 1808, for strangulated congenital hernia.*

---

* See an account of this case, drawn up by Dr. B. H. Coates, and published in the second volume of the North American Medical and Surgical Journal—also Dorsey's Surgery, vol. i, p. 96.

The sac being opened, the two coils of the bowel were found to be firmly adherent to the testicle, as well as partially to the abdominal ring, and one of them presented an opening of sufficient magnitude to permit the discharge of a considerable amount of fæces. There were, however, no marks of mortification, and the perforation was probably caused by ulceration. The symptoms were but slightly relieved by the division of the stricture, the patient continued very restless, and only a small quantity of matter flowed through the wound. Another operation was therefore performed, followed by much greater facility for the escape of the fæces. On the 24th of December, the projecting portion of the gut was cut off close to the ring, in the hope that the open orifices thus left would gradually retract within the abdomen. No good, however, resulted from this procedure, nor did any better success attend the method of Desault, which was employed soon after. It now occurred to Dr. Physick that relief might possibly be afforded by cutting a lateral opening through the sides of the gut; but not knowing to what extent they adhered to each other, he determined to pass a needle armed with a ligature from one cylinder to the other, about an inch within their respective orifices. This operation was performed on the 28th of January, 1809. The ligature, applied with moderate firmness, was secured with a slip-knot, and drawn to its original tightness whenever it became loose by the ulcerative action of the parts which it embraced.

After three weeks had elapsed, the ligature was removed and the parts in front of the opening which it had made divided with the bistoury. No unfavorable symptoms supervened upon this operation; on the 28th of February the patient had uneasy sensations in the lower portion of the abdomen, and on the 1st of March he extracted with his own fingers some hardened fæces from the rectum. Other evacuations followed; the discharge from the groin became inconsiderable; and the artificial anus gradually diminished in size. The patient was dismissed from the hospital on the 10th of November, in good health, but with a *fistulous* aperture in the groin, the hope of an entire closure being abandoned. By wearing a truss with a compress and a large pad, stuffed in the common way, the escape of fæces was completely controlled.

By the French surgeons the honor of the discovery of this process is generally claimed for Dupuytren, by whom it was executed at the Hotel-Dieu in 1813, without any knowledge apparently that it had been recommended by Schmalkalken, and performed by Physick.* His patient was Francis Aucler, thirty-six years old, of excellent constitution, who had been affected from his youth with an inguinal hernia on the left side, from which, however, he had experienced no inconvenience until the 13th of May, when, in consequence of a violent attack of vomiting, it became strangulated. Five days after he was carried, in a state of great prostration, to the Hotel-Dieu, and the stricture carefully divided. The bowel being sphacelated was left at the outer opening, and an artificial anus followed. Through this the whole of the fæces were discharged. Six weeks having elapsed without the prospect of a cure, Dupuytren employed compression, but violent symptoms ensued, and he was obliged to abandon it. He then resorted to the seton. The operation was soon over, gave rise to scarcely any pain, and was not succeeded by any accident. Some days afterwards a skein was substituted for the thread, when flatus began to be expelled by the natural anus. The size of the seton was increased at each dressing, and in less than eight days the patient had a passage from the rectum, preceded by colicky pains. In dressing the sore one day the buttress in front of the foreign body was completely lacerated, without any other effect than a more easy flow of fæces from one end of the bowel to the other. Stercoraceous matter continuing to escape by the abnormal aperture, Dupuytren excised by means of blunt-pointed scissors, directed on the fore-finger, half a line of the septum which intervened between the two openings. The operation was cautiously re-

---

* Leçons Orales, T. ii, p. 236. Paris. 1832.

peated at intervals of three or four days, the new adhesions were never passed, and the communication was soon so free that the fæces were evacuated entirely by the accustomed channel. Compression was kept up on the artificial anus, and the case was going on well, when Dupuytren, yielding to the entreaties of his patient, who was anxious to expedite the cure, divided the partition higher up than he had done before. In a few hours acute peritonitis supervened, and the man died. On dissection, a large quantity of serum and lymph were discovered in the abdominal cavity, but no stercoraceous matter, or any opening by which it could have found its way in. The communication between the extremities of the gut was re-established for the space of two inches, and instead of being separated, as they had been previously, they were united by a sort of raphé or cicatrice, the remains of the former septum. In fact, it clearly appeared that, but for the peritonitis, a complete cure would have ensued.

Discouraged by this unhappy termination, Dupuytren determined to abandon the employment of the seton, and to devise some other expedient. After many trials on animals and the dead body, he finally invented an instrument which he called the *enterotôme,* and which is thus described in the second volume of his surgical lectures. It consists of a screw and two branches, each about seven inches long. One of these, which is called the male branch, as it is received by the other, has a blade four inches in length, three lines in breadth, and half a line in thickness at its edge, which is undulated, and terminated by a spheroidal button. At the junction of the blade with the handle is a mortise several lines in extent; the handle itself is from two to three inches long, and has another mortise four lines broad, which runs nearly from one end to the other. The female branch is somewhat shorter. It is composed of two blades, of the same length, breadth, and thickness as the small blade, which is received in the gutter, groove, or sheath between them. The bottom of this gutter is undulated, to correspond with the

3 *

irregularities of the male branch, and at the extremity is a cavity for lodging the spheroidal button of the latter. At the union of the groove with the handle is a moving pivot, which passes into the mortise of the other branch, and the handle itself is terminated by a hole to receive the screw. This last part of the enterotôme, a screw of several threads, is an inch and a half long, and ends by an oval plate; it is placed in the mortise of the male branch, and fixed in the female, its use being to separate or close at pleasure the two blades.

The accompanying wood cut conveys an accurate idea of the nature of this instrument. *a* Represents the male blade, *b* the female blade, *c* the joint, *d* the moving pivot, *e* the handles, and *f* the screw by which the branches are shut and locked.

Before applying this instrument the surgeon satisfies himself of the precise situation of the lower opening of the gut, which it is by no means always easy to do, and places the patient upon his back near the edge of the bed, with the abdominal muscles completely relaxed. Taking one of the blades he conveys it upon the fore-finger into one of the orifices, to the requisite depth, and gives it to an assistant. The other is then introduced in the same manner into the other extremity of the tube, when they are joined together, like a pair of obstetric forceps, by putting the tenon of the one into the mortise of the other. The partition must be embraced to an extent of one, two, or three inches, according to the nature of the case; and the pressure, which is regulated by the screw, should be strong enough to destroy the vitality of the part the first few hours, as this will prevent pain and inflammation. It should afterwards be increased every forty-eight hours, until the enterotôme falls off, which it usually does from the seventh to the tenth day, along with the mortified portion of the partition. The operation is rarely attended or followed by any unpleasant symptoms, and the opening which it leaves generally affords free passage to the fæcal matter from one extremity of the bowel to the other.

From a statement published by Dupuytren in the work previously referred to, it appears that from the time he first employed this instrument until 1824, twenty-one operations had been performed by himself, and twenty by other practitioners. Three-fourths of the cases were caused by gangrene from strangulated hernia, and the remainder by penetrating wounds, with more or less loss of substance of the tube. Of the whole number thus treated three only died; one from supposed fæcal effusion into the abdomen, one from indigestion, and one from acute peritonitis. Of the thirty-eight that survived, none experienced any ill effects except a few who had colicky pains, nausea and vomiting, but were promptly relieved by effervescing draughts, leeches to the anus, and fomentations to the belly. The success was not equally great in all the cases. Twenty-nine were radically cured in from two to six months, and the rest retained, in spite of all that could be done, fistulous openings, which compelled them constantly to wear a compress and bandage, to prevent the escape of air, mucus, bile, and even fæces. "It would thus appear," says Dupuytren, "that the mortality from the use of the enterotôme is one in fourteen; or, if we exclude the case of indigestion, which cannot be fairly ascribed to the application of the instrument, it is reduced to one in twenty; a result much more favorable than that which usually attends the great operations of surgery."

One cause of failure of this operation, as was intimated in a previous page, is the want of adhesion in the sides of the opening made in the epéron, or septum. Velpeau relates the particulars of a case of this kind which fell under his own observation, and similar examples have occurred in the hands of other surgeons. His patient was a man fifty-six years old, who had a crural hernia of the left side since the age of eighteen: it was somewhat bigger than a hen's egg, became strangulated on the 17th of April, and was operated upon on the 27th of the same month. The intestine was found mortified, and the fæces soon after commenced passing through the wound. The enterotôme, applied on the 14th of May, was removed on the 21st, and the man expired on the 22d, death having been preceded by violent colicky pains, tympanites, and great tenderness on pressure of the hypogastric region. The lips of the two ends of the bowel were quite detached, and the margins of the opening in the intervening septum were adherent only on one side. Thus a free communication was established between the tube and the cavity of the peritoneum, which had become extensively inflamed from the contact of stercoraceous matter, and was filled with sero-purulent effusion.*

It can hardly be doubted that this case would have had a very different termination, had a longer period been allowed to intervene between the application of the enterotôme and the operation for the strangulated hernia. The attachments

* Velpeau, Medicine Operatoire, T. iv, p. 157.

between the ends of the intestine and the parietes of the abdomen were not sufficiently firm to resist the traction occasioned by the presence of the instrument, and hence the aperture which permitted the fæcal matter to pass into the abdomen. The want of union in the sides of the opening made by the enterotôme was probably caused by the unhealthy condition of the parts, which indisposed them to adhesive inflammation.

Some difference of opinion still exists respecting the propriety of closing the blades of the enterotôme on their first application so firmly as to destroy at once the vitality of the intervening septum. Dupuytren appears to have pursued this practice in all the cases which he treated with this instrument, without experiencing any ill effects from it. In the hands of others, however, the results have not been so successful. Jobert in particular objects to the plan on the ground of its liability to be attended with severe suffering. In all the cases witnessed by himself the patients were affected with fever, heat of skin, colicky pains, and vomiting; the countenance was livid and contracted, and the symptoms closely resembled those of strangulation. He refers to a case that occurred at the Hotel-Dieu of Amiens, were death was produced by the pressure of the enterotôme, and adds that examples of a similar description have been recorded by different practitioners.* He, therefore, advises that the instrument should be applied rather loosely in the first instance, and gradually tightened until it produces the desired effect. My own experience does not enable me to speak positively on this subject one way or another. In one case in which I employed the enterotôme the blades were applied with great firmness, and yet no unpleasant symptoms followed. The practice seems to me to be perfectly rational, and where bad effects follow, the pressure of the instrument may be diminished at any moment.

---

*Traité des Maladies du Canal Intestinal, T. ii, p. 126.

The following is an abstract of the first case in which Dupuytren employed the enterotôme on the human subject. Ménage, twenty-six years of age, was admitted into the Hôtel-Dieu, in January 1816, with an artificial anus in the right groin, produced by gangrene of the bowel twelve months previously. At first the evacuations were passed through the abnormal opening; but eight weeks after the operation, which had been performed for his relief, he was attacked with colicky pains, and had several natural stools, which afterwards recurred, though at long intervals. On his admission, the artificial anus was at least half an inch in diameter, and surrounded by irregular projections of the lining membrane of the gut, while behind a hernial protrusion appeared whenever he exerted himself, and frequently gave rise to invagination of the intestine. The skin around was raw and sore, the suffering severe, the stench intolerable. Having allayed the irritation of the parts, the blades of the enterotôme were separately introduced, as high as they would go, into each portion of the canal, and closed with considerable firmness. No pain was felt, and the next morning the pressure was increased, when slight colic was experienced. In a few days the blades became somewhat moveable. On the sixth, the man had several small evacuations by the natural outlet, and, on the eighth, the instrument fell off, holding in its grasp a membranous band, twenty lines in length by two in breadth. From this period the fæces passed by the rectum, but the artifical anus, though narrowed, still continued open, notwithstanding the employment of pressure, adhesive plaster, and lunar caustic. At length the edges were pared off, and brought together by the twisted suture, aided by a particular instrument. At the expiration of four months the patient was exhibited to the Faculty of Medicine, entirely cured of his infirmity.

Several surgeons, impressed with the conviction that the enterotôme of Dupuytren does not fulfil all the indications which may be expected from such an instrument, have endeavored to modify and improve it. It is not necessary to notice all these attempts; I shall glance only at a few of the more important, inasmuch as the principle is the same in all.

Liotard,* a French surgeon, recommends an enterotôme, the blades of which end each in an oval ring, an inch and a half in length by nine lines in width, and so constructed that the blunt crest of one is received in the corresponding gutter of the other. With this instrument he proposes to cut a hole *through* the septum, instead of destroying it from before backwards, as in the method of Dupuytren. He alledges that, when thus executed, the operation is followed by a free passage for the transmission of the fæces, and that the two extremities of the gut are more apt to regain their accustomed movements in the cavity of the abdomen. On the other hand, it has been urged that this instrument is not only difficult of introduction, but that it is liable to grasp a neighboring coil of the intestine or a fold of the omentum. This objection, however, if it is not entirely chimerical, as I am disposed to believe it is, is no more applicable to this contrivance than to the original; which, as has been seen, often embraces the septum to the extent of two, three, or even four inches, without any ill effects. The only objection that I can perceive is, not against the enterotôme itself, but to the manner in which it is used. The portion of the partition left undivided in front, or between the outer orifice and the opening of communication, might possibly interfere with the reparative process, but I am not certain that it would even do this. That it would prevent the cicatrization of the sore, and predispose to the formation of a permanent fistula, as some have pretended, is not very probable.

The only instance in which, so far as my information ex-

---

* Diss. Sur le Traitement des Anus Contre Nature—Dict. de Medicine, T. iii, p. 365—Jobert, op. cit. T. ii, p. 129—Malgaigne, Med. Operat. p 572.

tends, the enterotôme of Liotard was used on the human subject, has been recently related by Blandin.* The disease, in this case, was caused by strangulated inguinal hernia, in which six inches of intestine had become gangrenous. The two extremities of the tube lay parallel with each other; the fæcal matter escaping from the superior, which was very tumid externally, and readily admitted the finger. The inferior one was more contracted, and its diameter daily diminished. The superior end formed an irreducible tumor, which Blandin comprehended in a ligature, and it sloughed off on the fourth day. He then constructed an enterotôme composed of two branches, each of which terminated by an oval ring, from eighteen to twenty lines in length, and from six to eight in breadth; the internal surface being marked by alternate elevations and depressions. This instrument was introduced into the extremities of the bowel, to a depth of four or five inches, and compressed by means of a screw. Abstinence and rest were enjoined; no bad symptoms ensued; and the enterotôme separated on the fifth day. On the same evening the patient voided solid fæces by stool for the first time during an entire month. Gas and a yellowish green fluid continued to escape for sometime at the external orifice; but this gradually ceased, and the cure was completed in two months after the employment of the instrument.

Another modification of the enterotôme was proposed by the late Professor Delpech of Montpelier.* He was of opinion that the original instrument divided the partition to too great extent at one time, and that the application of it was liable to be followed, from the contact of fæcal matter, by protracted suppuration, together with ultimate contraction of

---

* Archives Generales de Medicine, T. xii, Nov. 1836.—British and Foreign Med. Review, vol. iii, p. 520.

* Observations sur l'Anus Artificiel; Mémorial des Hôpitaux du Midi, Février, 1830, p. 76.

the parts, and a partial re-production of the ridge. These inconveniences he thought might be obviated by the more gradual division of the intermediate structures; and for this purpose he devised an instrument fashioned somewhat like a compass, with thin, hollow branches, slightly curved, and terminating each in a sort of spoon, an inch long, and provided with a blunt rim. The branches are united by a screw, and are introduced separately into the bowel upon an ebony gorget. The enterotôme is then locked and secured by a thread or tape to a bandage round the corresponding thigh. When the spoons are applied it is said that they embrace the septum to the extent of *four inches in depth by upwards of an inch in breadth.** Nevertheless, Delpech considers this instrument a valuable improvement, and has published a case in which its employment was followed by complete recovery.

The most important modification, perhaps, of the enterotôme, if indeed it may not be regarded as an entirely new instrument, was suggested in 1827 by Reybard.† It is difficult to convey an intelligible idea of it without the assistance of a drawing. In its general form it resembles a pair of dissecting forceps, slightly curved on the surface for two inches, or to within a short distance of its junction with the branches, which are themselves four inches long, and rounded off at the end. The branches are, moreover, flattened in their entire extent, and fenestrated in the same direction from their origin to within two or three lines of their extremity. Near the handle, on each side of the gutter or slit-like opening just alluded to, are two screws by which the instrument is closed and locked. Introduced into the upper and lower end of the gut, they firmly hold and compress the septum, without contusing it, or depriving it of its vitality. Adapted to the fenestra of the upper branch is a moveable knife, designed

* Dict. de Médecine, T. iii, p. 365.

† Mémoires sur le Traitement des Anus Artificiels, et des Plaies des Intestines, Lyon et Paris, 1827.

for dividing the partition to the extent of two or three inches. This section would be attended with hemorrhage, were it not for the pressure exerted by the instrument, which is kept on for about forty-eight hours, or until the adhesions in the adjacent structures are sufficiently firm. In two cases in which Reybard employed his enterotôme, the operation produced hardly any pain, no bad symptoms ensued, and the patient rapidly recovered.

Finally, a new enterotôme, regarded by some American surgeons as a valuable improvement on that of Dupuytren, was invented in 1835, by Dr. J. R. Lotz, of New-Berlin, in the State of Pennsylvania.* The blades, which are six inches in length, terminate each by an oval fenestra, twelve lines long by three in breadth, and surrounded by a narrow, solid rim. They are articulated in the following manner. At the upper extremity, or that which in forceps answers to the joint, and also at the middle of one of the blades, are two steel slides, which are fitted into mortice holes in the corresponding parts of the other blade. Near each of these slides is a screw, of which the posterior passes through one blade, and simply presses on the other, to regulate the distance between them, while the one at the centre of the instrument extends through both blades, approximates them, and presses the edges of the fenestræ against each other.

The mode of applying this instrument does not differ from that of Dupuytren. The blades being unscrewed, and inserted separately into the intestinal orifices, the two slides are introduced into the mortice holes, and the fenestræ brought upon a line with each other. "The central screw is now introduced, and the adjusting screw having been previously turned far enough to allow for the thickness of the double walls of the intestine included between the pinching extremities, the central screw is tightened until the edges of the fenestræ press firmly upon the intervening membranes. By unscrewing the adjusting screw and tightening the central

---

* American Jour. Med. Sciences, vol. xviii, p. 367.

one, the pressure can be increased to any requisite degree without destroying the parallel direction of the blades."

Dr. Lotz, I believe, has employed this instrument only on one occasion. His patient was a woman forty-one years of age, and the disease, caused by a gangrened hernia, had existed four or five weeks. On the fourth day after the operation, he excised, with a gum-lancet, the portion of the septum corresponding with the fenestræ, and established a direct communication between the two ends of the tube. The instrument was now gradually slackened, and in a week it was removed altogether. On examining the parts, a smooth circular hole was found, about the dimensions of an inch, with the bowel firmly adherent all around. When the case was reported, several months after the operation, the fæces passed nearly all by the natural channel, and the patient was far advanced in utero-gestation. Whether complete recovery ultimately ensued, I am not able to state, as no further account of the case has, I believe, been published.

Having had occasion recently to treat a case of artificial anus, it occurred to me that an enterotôme might be devised, much more simple, and in all respects much better adapted to the purpose, than any of those noticed in the preceding pages. Accordingly with the assistance of Mr. Erringer, an ingenious cutler of this city, I had an instrument constructed, of which the following is a description. In its general appearance, it closely resembles an artery-forceps, being composed of two blades, and of an intermediate catch. Each blade is five inches in length, and terminates anteriorly in an oval ring, eighteen lines long by eight in width. In thickness the ring does not exceed the twelfth of an inch; it is smooth and convex externally, but the inner surface is undulating, or marked by alternate elevations and depressions. The catch by which the blades are closed is situated at the centre of the enterotôme, and is furnished with a rack, for the purpose of regulating the amount of pressure when it is applied to the epéron between the two ends of the bowel. The weight of the instrument is scarcely half an ounce.

*fig.* 1.

*fig* 2.

The annexed drawing, prepared by my friend Dr Bayless, will explain the nature of this new enterotome more satisfactorily than any description, however elaborate. Figure 1, represents the two blades, the manner in which they are connected behind, and the rings in which they end in front. When they are closed, the depressions of one ring receive the elevations of that of the other, and thus prevent the instrument from losing its hold. Figure 2, exhibits the catch and the arrangement of the teeth of the rack.

The instrument here described and delineated, I have not had an opportunity of employing upon the human subject. In the case adverted to I used an enterotôme of a more clumsy construction, the blades of which, connected by a hinge-like joint, were closed by a central screw, and terminated each in an oval ring, about twelve lines long by eight in width, and perfectly smooth at the inner surface, instead of being rough and undulating, as in the new instrument. It was applied with great firmness, and dropped off at the end of the sixth day, including the portion of the septum which it had embraced. No bad symptoms followed the constriction. For the first few hours there was some uneasiness in the part, and the patient, a colored boy, sixteen years of age, complained of slight nausea. These, however, rapidly subsided, and did not subsequently recur. As the case is still under treatment, I shall content myself with the following abstract of it, intending to present a more full report at some future period.

In December, 1840, as I learn from Dr. Ford, of Somerville, Tennessee, the boy was caught in the machinery of a cotton-gin, by which the parietes of the abdomen were torn open at their lower part; the wound extending, on the one hand, from the pubic symphysis to within an inch of the umbilicus, and, on the other, from a short distance above the anterior superior spinous process of the ilium of one side to that of the other, forming a large flap, which was drawn up so as to expose the small bowels. The accident was attended with very little hemorrhage. Dr. Brackin, who saw the boy soon afterwards, brought the edges of the wound together by stitches and adhesive straps, aided by a suitable bandage. Perfect quietude was enjoined, and the most rigid antiphlogistic regimen adopted. After some days the flap alluded to sloughed off, when it was ascertained, for the first time, that the small bowel had sustained some injury. In a few months the opening had contracted to about one-fourth the original size, the intestine adhered firmly to the margin of the artificial anus, and the passage of fæcal matter was easily controlled by a compress and bandage. Several attempts were subsequently made to close this outlet, but they all proved unsuccessful.

When the boy was sent to me, last March, the opening, situated on the left side of the median line, nearly midway between the groin and the umbilicus, was of an oval shape, two inches in its transverse diameter, and fifteen lines in the vertical. The surrounding parts had a raw, excoriated appearance, and the bottom of the ulcer was formed by the two ends of the bowel, lying parallel with each other, the upper orifice being external, the inferior internal. The sore was nearly half an inch in depth; the edges were bevelled, or depressed towards the centre, and nearly all the fæces flowed in this direction, except when the parts were protected by a compress and bandage. When in the erect posture, there was, at times, a considerable protrusion of the mucous membrane, especially of that of the lower end of the intestine. The intervening septum was well-marked, and formed a serious obstacle to the transit of the fæcal matter from one orifice to the other. The boy's appetite was good, and his general health unimpaired.

Having destroyed the intervening septum by means of the instrument above alluded to, the fæcal matter soon passed, in great measure, along the natural route; but there was no disposition whatever in the opening to contract, owing to the loss of muscular substance. Under these circumstances, I pared away the edges of the protruded mucous membrane of each end of the bowel, and approximated them by several points of the interrupted suture. In this manner I succeeded in obtaining a considerable degree of union; and by repeating the operation, as has been since done a number of times, the opening is so far closed that there is now, June the 20th, only a small slit-like aperture at each angle of the ulcer. By perseverence I shall be able, I think, to effect complete adhesion between the parts, and relieve my patient of his loathsome disease.

In 1820, Mr. Collier,* a surgeon of London, conceived and executed the plan of curing an artificial anus by a sort of *autoplastic* operation. The patient was a male servant, and the aperture, occasioned apparently by a stercoraceous abscess, occupied the right groin, being large enough to admit two thumbs. When Mr. Collier first saw him, two or three months after the formation of the artificial anus, it had considerably diminished in size, and was surrounded by callous edges. When the bowels were constipated the fæces escaped chiefly by the abnormal opening, but when they were relaxed,

* London Medical and Physical Journal, vol. lxiii, p. 466.

or under the influence of medicine, nearly all came away by the natural route.

After several failures to unite the parts by suture and other means, Mr. Collier determined, if possible, to effect a cure by an operation on the principle of Taliacotius. With this view, having pared away the indurated margins of the sore, and converted them into a raw surface about twice the diameter of the artificial anus, he dissected off an adequate cutaneous flap immediately above it, and placing it over the aperture, maintained it by four stitches. A compress and truss constituted the dressings. Complete recovery ensued, without any unpleasant symptoms.

A similar operation was lately executed, with the same happy results, by Mons. Blandin. An account of this interesting case will be found in the Memoirs of the Royal Academy of Medicine of Paris, for 1838, and also in the "Gazette Médicale" for July of the same year. The patient was a countryman, aged fifty-two, and the artificial anus, produced three years previously by a gangrened rupture, was situated in the inguinal region of the right side where it communicated with the cœcum. Nearly all the fæces passed out at the abnormal opening, which was of a circular shape, and sixteen lines in diameter. There was occasionally also a considerable protrusion of the bowel. After the failure of the more ordinary methods of treatment, a quadrilateral flap of the skin and cellular substance was made just below the opening, by three distinct incisions, and dissected up for the space of nearly two inches, being left adherent by one of its edges to the inguino-crural region. The integuments over the superior internal and external margins of the opening were then removed to the extent of about three lines, when the flap was drawn up and put in contact with the parts, with its bleeding surface looking backwards, and the points of suture placed beyond the circle of the artificial anus. A piece of linen spread with cerate, a layer of charpie, a few compresses, and a truss with a weak spring, formed the dressings. By the fourth day the flap had perfectly united, both externally and internally, except superiorly, where it became necessary at the end of a fortnight to pare away the opposing edges, and bring them together by the twisted suture. The adhesion after this operation was almost complete, two little holes only remaining, and these speedily cicatrized under the use of the nitrate of silver. The walls of the abdomen were preternaturally feeble at this point, and required to be supported by a truss.

This plan has likewise been tried by Velpeau, but without success. On the second day a yellowish serosity with stercoraceous air was observed to escape between the sutures, and the flap was seized with gangrene extending from its edges towards the pedicle by which it adhered to the neighboring parts. The operation of Collier appears to be more particularly applicable to those cases of the disease which are unattended with an intervening septum, and in which the gut retains its normal caliber, or nearly so. It will also be more likely to succeed where the outer orifice is small, and the margin soft and healthy. Under opposite circumstances it can hardly be attempted with the prospect of a favorable result.

Another method, somewhat analogous to the preceding, was put in practice a few years ago by Velpeau.* It consists in making two semi-elliptical incisions around the preternatural aperture, about fifteen lines exterior to it, and in approximating the parts, previously made raw, by several points of suture. The new wounds are filled with charpie, and the dressings are completed by the application of a compress and bandage. The ligatures, which are all introduced before making the lateral incisions, are tied with moderate firmness, and carried through the parts in such a manner as to prevent them from tearing out, or injuring the bowel or peritoneum.

This new operation was executed by Velpeau, for the first time, on the 15th of November 1835. His assistant was Dr. Mott, of New York, and the patient a young man from Nor-

* Médecine Opératoire, T. iv, p. 153.

mandy. As a preliminary step, he pared away the margins of the abdominal aperture, obliquely from the circumference towards the centre, and in such a manner as not to injure the gut, or its mucous membrane. He next introduced four sutures, at intervals of two lines from each other, and with the precaution of not penetrating the abdomen or the intestine. A semi-lunar incision, two inches long, and including the skin, cellular substance, and aponeurosis of the external oblique muscle, was now made around each side of the artificial anus, twelve or fifteen lines beyond it. The parts being cleansed, the operator tied the sutures, and laid a roll of charpie in each lateral wound, to separate its edges, before he applied the dressings.

Three days after the operation there was such an accumulation of alvine matter as to render it necessary to cut away the sutures. The edges of the sore were washed, and the patient, kept as quietly as possible, was allowed a more liberal diet. On the 30th of December the purulent discharge had almost ceased, and by the 4th of January 1836, the fæces had entirely resumed their accustomed route. To render the cure more certain, the patient remained in the hospital until the 8th of February.

The object of this ingenious operation, as will be readily perceived, is to bring the raw margins of the outer orifice in contact with each other, to facilitate their re-union. The artificial anus is thus converted, to use the language of Velpeau, into a sort of basin, the bottom of which is sensibly smaller than the entrance or mouth. The approximation, easily accomplished by the lateral incisions above described, has the effect of completely closing the intestinal portion of the orifice. A strict regimen, perfect quietude, and a gentle enema every evening, will promote the cure.

In 1827 Mons. Colombe,* a French surgeon, suggested the idea of re-establishing the continuity of the two extremities of the bowel by means of a large gum-elastic tube, slightly

---

* Biblioth. Méd. T. i, p. 389, 1827.

curved, and from two to three inches in length. This is introduced, first, into one end and then into the other, with the concavity resting against the epéron or septum, and secured by a ligature, passed through its anterior wall, to the outside of the abdomen. It is retained in this situation until the passage for the fæcal matter is sufficiently restored, and the external aperture nearly closed, when it is removed, and the case managed in the usual manner. This method was tried by Velpeau* in 1831, but his patient died three days after under symptoms of acute peritonitis. The intestine had been perforated at its posterior part, and the tube projected across the opening, but whether as cause or effect, could not be ascertained.

Another proceeding which has been lately proposed is *excision* of the intervening septum. The operation was first performed, a few years ago, by Mons. Raye, a French surgeon. He seized hold of the epéron or partition between the two ends of the bowel with a pair of polypus-forceps, and cut out a large V-shaped flap with the scissors. No untoward symptoms followed the operation, and the patient promptly recovered. The particulars of this interesting case will be found in the "Gazette Médicale" for 1838.

The artificial anus is occasionally found to open into the vagina, as in the interesting cases mentioned by Casamayor and Roux. In the first of these the posterior wall of the vagina was lacerated during a severe and protracted labor. A fold of the ileum, lying in front of the rectum, was forcibly compressed during the descent of the child's head, and, although it contracted firm adhesions to the tube in question, it became subsequently, like the rest of these parts, involved in gangrene; the deplorable disease alluded to was the consequence. To remedy this, Casamayor endeavored to establish a communication between the ileum and the rectum, so as to divert the fæcal matter from the vagina, which was nearly obliterated by the wound or rent, and make it pass directly

* Médecine Opératoire, T. iv, p. 153.

from one of these intestines to the other. He accordingly constructed a pair of forceps, six inches and a half long, the blades of which were about the diameter of a large quill, and curved in such a manner as to intercept a free space at their base. Anteriorly they terminated each by a ring eight lines long by four in breadth. The patient being placed upon her back, one of the blades was carried along the vagina into the small bowel, about an inch and a half above the abnormal opening, and the other to the same height into the rectum. Having ascertained that the enterotôme embraced nothing but the opposed parietes of the intestines, the blades were approximated and locked. No evil consequences ensued. The instrument was removed six days after its application, leaving above the intestino-vaginal orifice an opening through which the fæces flowed directly from the ileum into the rectum. From this time the natural stools began to be re-established, a small quantity of matter only passing along the vagina. Unfortunately at this period the woman was attacked with a violent inflammation of the lungs and pleura, of which she died in four days.*

In the case related by Roux the procedure was entirely different. After breaking up the adhesions between the bowel and the vagina, he attempted to unite the two ends of the intestinal tube by suture; but the woman soon died from the effects of the operation.†

---

* Dict. de Méd. et Chir. Pratiques, T. iii, p. 169.

† Dict. de Médecine, T. iii, p. 371.

## Figures and Tables

**Figure f1:**
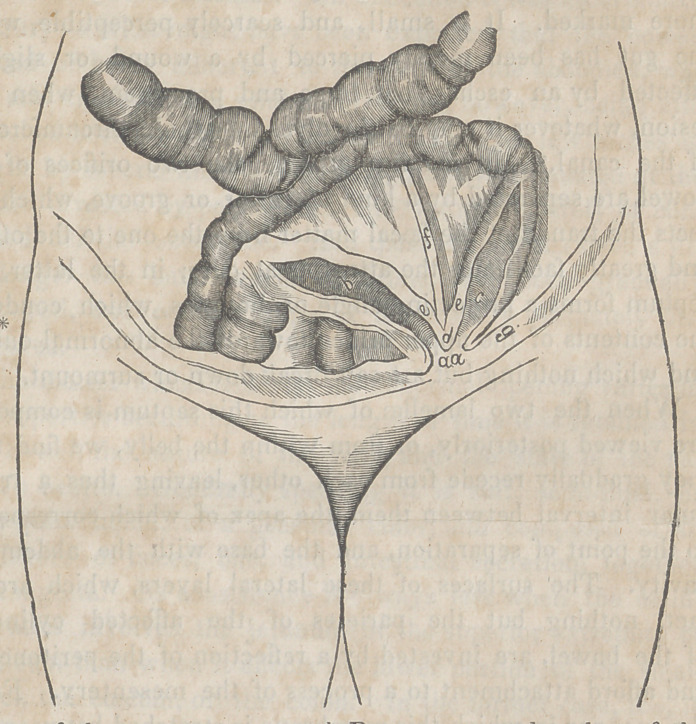


**Figure f2:**
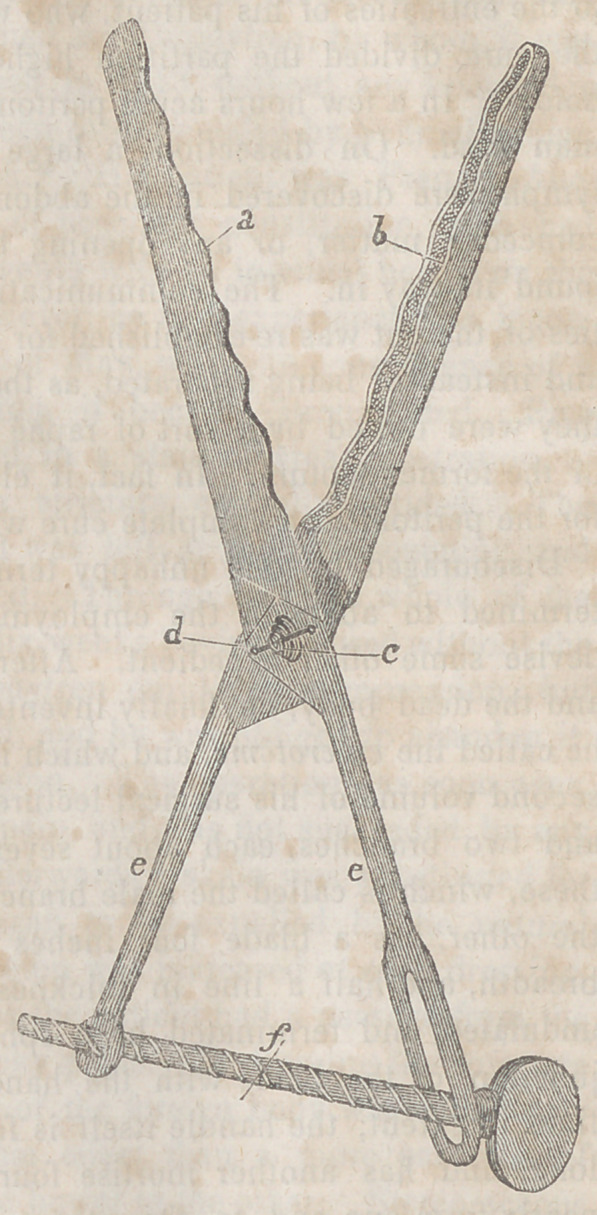


**fig. 1. f3:**
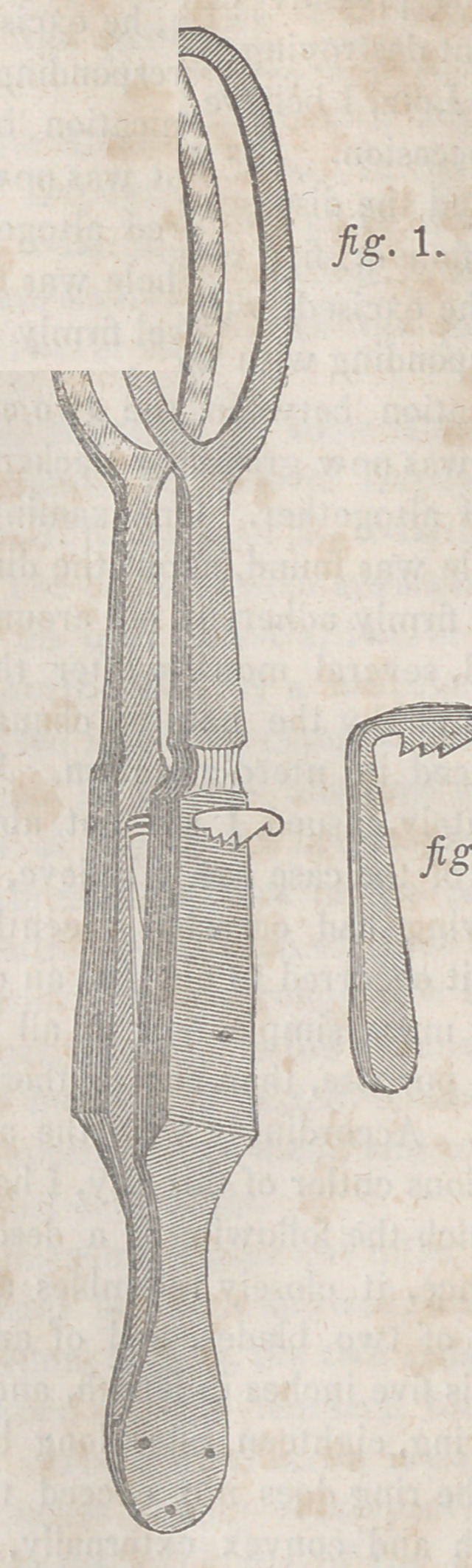


**fig 2. f4:**